# Intrathecal antagonism of microglial TLR_4_ reduces inflammatory damage to blood–spinal cord barrier following ischemia/reperfusion injury in rats

**DOI:** 10.1186/1756-6606-7-28

**Published:** 2014-04-21

**Authors:** Xiao-Qian Li, Jun Wang, Bo Fang, Wen-Fei Tan, Hong Ma

**Affiliations:** 1 Department of Anesthesiology, First Affiliated Hospital, China Medical University, Shenyang 110001, Liaoning, China

**Keywords:** Blood–spinal cord barrier, Intrathecal transplantation, Microglia, Minocycline, Toll-like receptor 4, Spinal cord ischemia/reperfusion injury

## Abstract

**Background:**

Inflammatory reaction in blood–spinal cord barrier (BSCB) plays a crucial role in ischemia/reperfusion (I/R) injury. It has been shown that microglia could be activated through Toll-like receptors (TLRs). Therefore, we hypothesize that TLR4 is involved in the microglial activation and BSCB disruption after I/R.

**Results:**

To verify our hypothesis, we analyzed the behavioral data, changes of BSCB permeability, as well as expressions of microglial marker Iba-1 and TLR_4_ in spinal cord I/R model induced by 14 min aortic occlusion. Double immunostaining reveals that after I/R, Iba-1 immunoreactivity increased gradually 12 h after reperfusion and maintained at a such level throughout 36 h. Such increasing pattern of Iba-1 expression is consistent with the increases in Evan’s Blue (EB) extravasation, spinal water content and mechanical allodynia demonstrated by lowed withdrawal threshold to Von Frey filaments. Moreover, double immunostaining suggested that TLR_4_ was highly expressed in microglia. Intrathecal infusion of minocycline and TAK-242 (TLR_4_ inhibitor) treatment attenuated I/R-induced allodynia and BSCB leakage. In contrast, LPS induced TLR_4_ expression aggregated above-mentioned injuries. Furthermore, the nuclear factor-kappa B (NF-κB) activity has a similar profile as TLR_4_ activity. It is consisted with the results of NF-κB mRNA and protein expression changes and activation of downstream cytokine, IL-1β. Expectedly, intrathecal infusion of pyrrolidine dithiocarbamate (PDTC), a NF-κB inhibitor, showed similar protective effects as minocycline and TAK-242. In addition, our data show that TLR_4_ closely involved in I/R-induced inflammatory damage induced neuronal apoptosis. Significantly, neutralizing TLR_4_ function largely reduced neuronal apoptosis determined by NeuN immunoreactivity in ventral gray matter and increased percentage of double-label cells with cleaved caspase3, whereas LPS reversed these effects. Similarly, inhibitions of microglia and NF-κB with minocycline or PDTC treatment accordingly perform the same protective effects on I/R injury.

**Conclusion:**

The results indicate that compromised BSCB caused by I/R injury lead to spinal microglial activation and TLR_4_, its membrane-bound receptor, up-regulation, which then initiate neuro-inflammation and neuro-apoptosis via NF-κB/ IL-1β pathway. To inhibit the positive feedback loop of TLR4-microglia-NF-κB/ IL-1β pathway by minocycline, TAK-242 (TLR_4_ inhibitor) and pyrrolidine dithiocarbamate (PDTC, NF-κB inhibitor) may provide new targets for treating I/R injury in clinic.

## Background

Spinal cord ischemia/reperfusion (I/R) injury is the most devastating complications encountered in many pathophysiological situations, such as hypotension, surgical procedures on thoracic, thoracoabdominal aneurysms and the spine [1]. It remains as a widespread and persistent problem, because its debilitating injuries to the central nervous system results in high incidence of paraplegia posing a serious threat to patients. However, the underlie mechanism of spinal cord I/R injury is not well understood. This is probably due to the induction of spinal cord I/R injury is multifactorial [2-4]. Blood–spinal cord barrier (BSCB), surrounded by astrocytes and perivascular microglia, consists of a continuous capillary endothelium with tight junctions between the cells. As shown in our previous studies, BSCB disruption and inflammatory reactions play an important role in the evolution of spinal cord I/R injury and in promotion of neuronal damage [5,6]. Research has expanded into the glial/neuronal transmission and immune responses of resident glial cells to spinal cord injury [7,8]. Existing evidence shows that spinal glial activation involves important components of the immune system and triggers rapid signal transduction cascades of the transcriptionfactor nuclear factor κB (NF-κB), driving gene expression of proinflammatory cytokines (e.g. IL-1β) in the course of pathophysiological changes that occur after brain injury [9]. Nonetheless, the specific cellular source within microglia which is responsible for transferring immune stimuli into the nervous system responses is unknown. During the pathogenic cascade after central nervous system (CNS) injury, microglia are thought to be the first nonneuronal cells to express a plethora of growth factors, chemokines, and regulatory cytokines as well as free radicals and other toxic mediators [7,10].

In microglia, Toll-like receptors (TLRs), especially TLR_4_, have been shown to recognize various microbial products and to initiate innate immune responses upon interaction with infectious agents or endogenous ligands present in the spinal cord *in vivo* and *in vitro*[10,11]. The general understanding is that TLR_4_ can be specifically activated to initiate immune responses by presenting a pathogen-derived antigen to naïve T cells when sensing lipopolysaccharide (LPS), a common constituent in the cell wall of Gram-negative bacteria [12].Studies have demonstrated that TLR_4_ is upregulated in different I/R-injured organs, including heart, brain, liver, and kidney [13,14]. Nevertheless, the relationship between TLR_4_ and microglial activation in spinal cord I/R injury remains unknown. Thus, we hypothesized that there might be a link between TLR_4_ of microglia to the stressors that damaged neurons: endogenous antibodies or cytokines that leaked through disrupted BSCB, which, in turn, contributes to further activation of microglia and BSCB disruption. Moreover, recent studies suggest that microglia activation which could be inhibited by minocycline is a double-edged sword in various neurological models and under different conditions [8,15]. Ischemia was regarded as a powerful stimulus that disabled the endogenous inhibitory signaling and triggered microglial activation [7]. Upon activation, microglia could exhibit plenty of phenotypes and release both pro- and anti-inflammatory mediators to either exacerbate ischemic injury or help repair. A few studies, however, have conducted on the spinal cord model of I/R injury and state that whether activation of microglia is deleterious and/or beneficial for spinal cord recovery is still a controversial topic. To testify our hypothesis, we first explore whether microglia are activated in a rat model of spinal cord I/R injury. Next, we evaluate the roles of TLR_4_ and NF-κB during I/R-induced BSCB disruption with and without the involvement of microglia.

## Results

### Alterations in the neurological deficit after I/R

**Figure 1:**
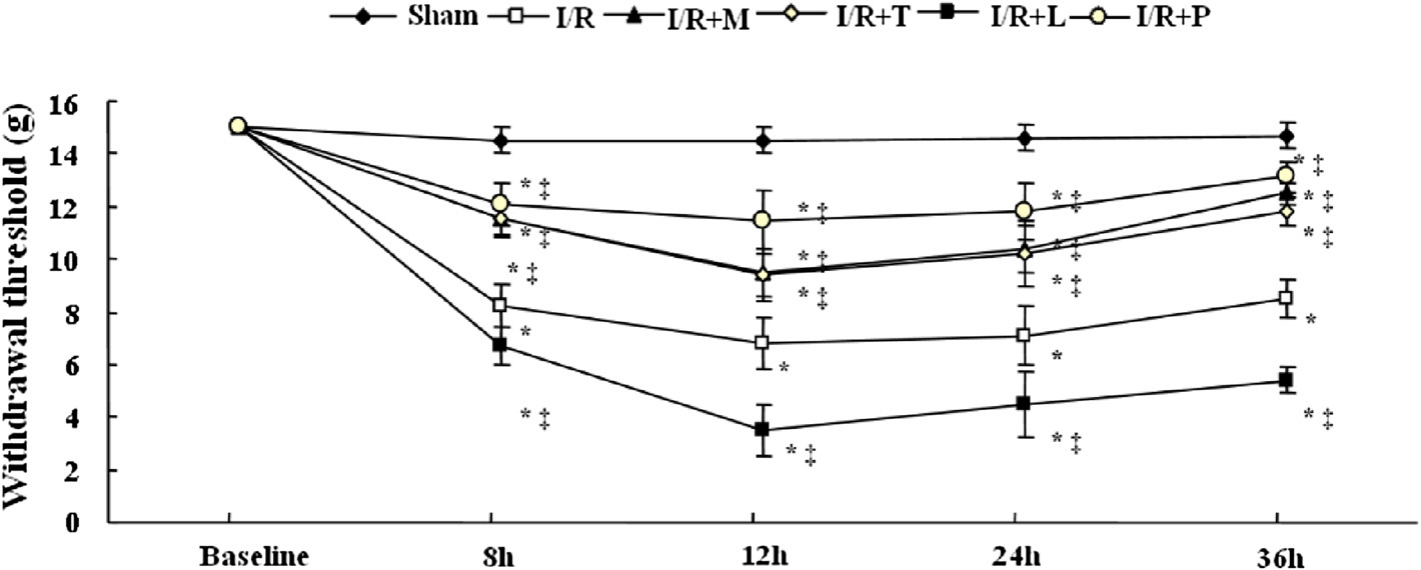
**Alterations in neurological deficits in mechanical sensitivity to von Frey filaments after spinal cord ischemia reperfusion (I/R) injury.** The significant decreases relative to baseline in withdrawal threshold (WT) on postoperative 8 h to 36 h in I/R group, suggesting that spinal cord I/R injury induced mechanical hyperalgesia in the paws. Continuous intrathecal administration of minocycline, TAK-242 and pyrrolidine dithiocarbamate (PDTC) for 3 days before the surgical operation increased the withdrawal threshold at time points 8, 12, 24, and 36 h compared to the I/R group. Intrathecal administration of LPS synergistically enhanced the mechanical hyperalgesia. **P < .01 compared to Sham group; ^##^*P* < .05 compared to I/R group in one-way analysis of variance followed by Tukey’s test (n = 12 per group).

As illustrated in Figure 1, the weights of rats significantly decreased relative to baseline in withdrawal thresholds (WTs) to Von Frey filaments throughout 36 h after reperfusion in I/R group (*P* < .05), indicating the development of mechanical allodynia is induced by 14 min thoracic aortic occlusion. Moreover, WTs of I/R group were much lower than those of intrathecally administrated minocycline treated group (I/R + M group), TLR4 inhibitor treated group (I/R + T group) and PDTC treated group (I/R + P group). On the other hand, WTs of I/R group were higher than those of LPS induced group (I/R + L group) throughout all observed time points postoperatively (*P* < .05). There were no significant differences between I/R + M group, I/R + T group and I/R + P group at above time points (*P* > .05).

### Effects of I/R on BSCB permeability

**Figure 2:**
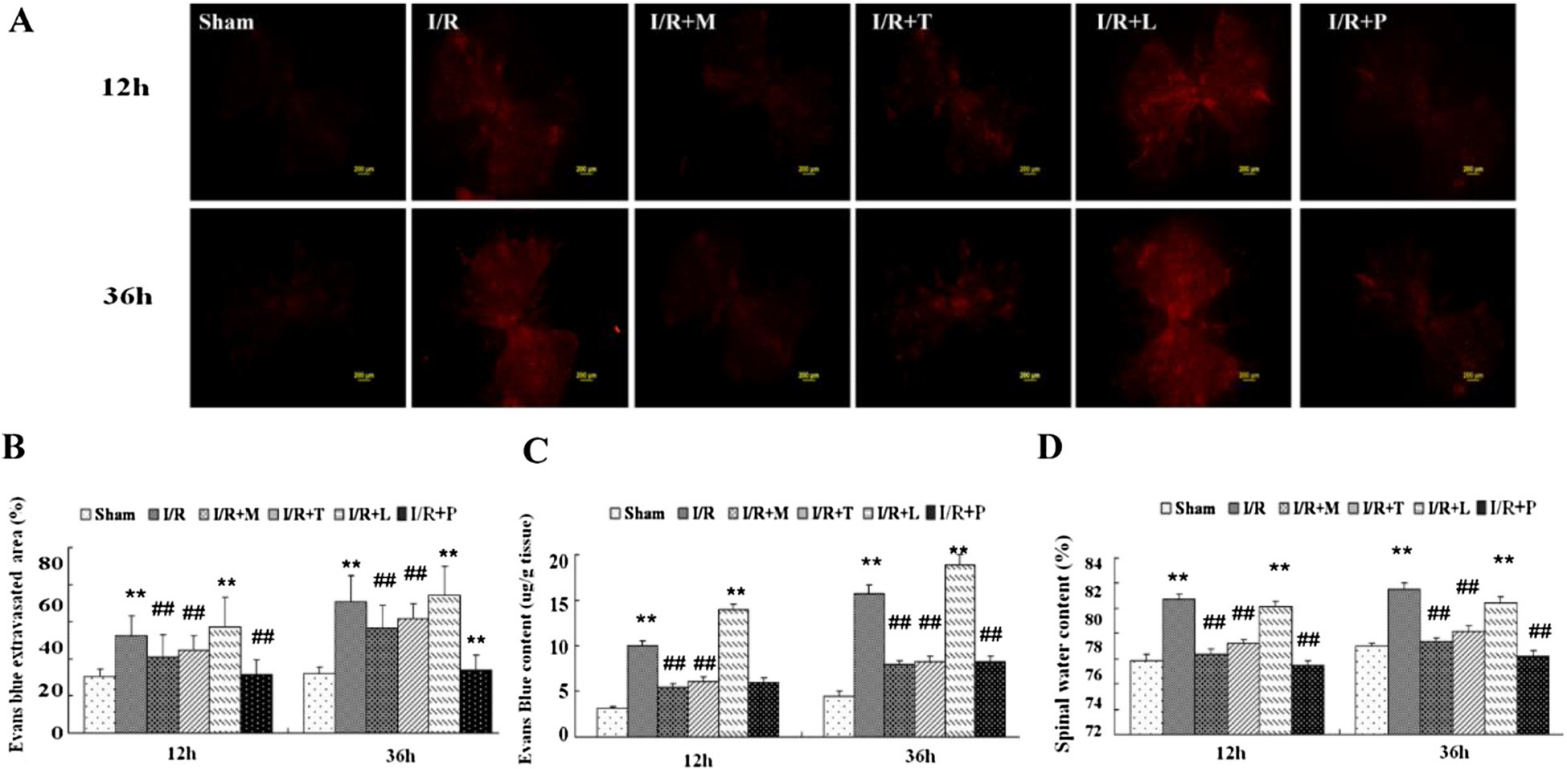
**Alterations in blood–spinal cord barrier (BSCB) integrity after spinal cord ischemia reperfusion I/R injury. (A)** Effects of spinal cord I/R injury on BSCB permeability measured by Evans blue (EB) extravasation. Almost no red fluorescence was seen in spinal cord of Sham group at 12 and 36 h after injury. Much more red fluorescence, especially in the gray matter could be seen in I/R group at 12 h after injury, which was even stronger at 36 h postoperatively. And EB red extravasation was significantly weakened in the groups pretreated with minocycline, TAK-242 and PDTC, whereas EB extravasation was worsen by intrathecal injection with LPS at above time points. **(B) **Percentage of EB extravasated area. **(C)** EB content of the spinal cord (μg/g). **(D)** Quantification of the water content of the spinal cord (edema). All data are presented as mean ± SEM (n = 8 per group). Scale bars are 200 μm. **P < .01 compared to Sham group; ^##^*P* < .05 compared to I/R group.

As shown in Figure 2*A* and *B*, BSCB permeability was changing in response to spinal cord I/R injury. BSCB permeability is visualized by extravasation of the Evans blue (EB) dye. I/R induced marked increase in the amount of EB extravasation comparing to the sham groups at 12 and 36 h after reperfusion (*P* < .01). Intrathecal infusion of minocycline, TAK-242 and PDTC (to suppress microglial activation, TLR_4_ and NF-κB function, respectively) reduce the EB content and the percentage of strained area at 12 and 36 h after the surgical procedure (*P* < .01 compared to the I/R group). In contrast, intrathecal infusion of LPS, a TLR_4_ agonist, synergized the BSCB leakage as the increased EB content and the percentage of strained area (*P* < .05 compared to I/R group). Quantification of the EB content of the injured spinal cord in Figure 2*C* confirmed that BSCB leakage induced by I/R injury was synergistically increased by LPS (*P* < .05 ) and was attenuated by minocycline, TAK-242 and PDTC treatments (all *P* < .01). There were no detectable differences in EB extravasation between the groups of I/R + M, I/R + T and I/R + P at all above time points (*P* > .05).

Furthermore, assessment of water content showed similar results as indicated in Figure 2*D*. I/R increased water content due to spinal cord edema (*P* < .01), and intrathecal infusion of minocycline, TAK-242 and PDTC markedly attenuated this effect at 12 and 36 h (*P* < .05), whereas treatment with LPS synergistically increased spinal cord edema (*P* < .05) .

### Effects of I/R on Microglial reaction in the spinal cord

**Figure 3:**
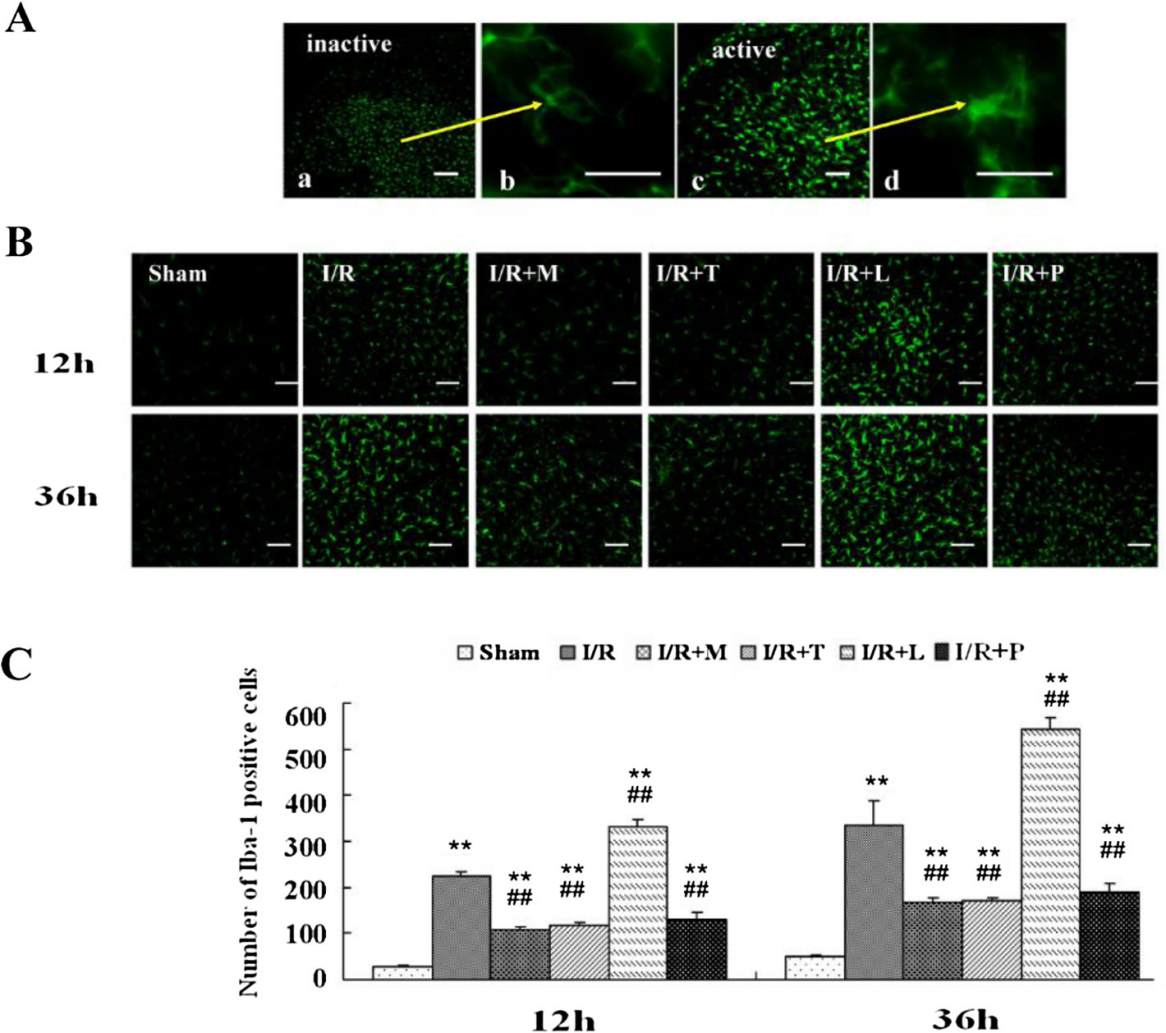
**Alterations in microglial reaction after spinal cord ischemia reperfusion (I/R) injury. (A)** Effects on the morphology changed in microglia after spinal cord after I/R injury. Scale bars are 200 μm in 3*A* a, c; 50 μm in 3*A*-b, d; **(B)** Effects on spinal immunoreactivity to Iba-1 after I/R injury. Scale bars are 200 μm. **(C)** Quantification of Iba-1–positive cells in the spinal cord’s dorsal horn is presented as mean ± SEM (n = 6 per group).Prominent Iba-1 positive cells activated by I/R injury were observed in both spinal dorsal horns of rats subjected to the surgical operation, who exhibited hypertrophic morphology with thick processes. Increases in Iba-1 immunoreactivity and number of Iba-1–positive cells in spinal dorsal horn were markedly attenuated by intrathecal injection of minocycline, TAK-242 and PDTC but was synthetically activated by LPS. **P < .01 compared to Sham group; ^##^*P* < .05 compared to I/R group.

As shown in Figure 3*A* and *B*, the fluorescence intensities of ionized calcium–binding adaptor molecule 1 (Iba-1) are commonly used to quantify activated microglia. Upon activation, microglial cells transform from the ramified shape (Figure 3A-a, b) to a rounded (amoeboid) macrophage-like shape. As shown in our study, typical activated microglia with Iba-1 staining exhibited hypertrophic morphology, with thick processes which could easily be distinguished from the inactivated ones ( Figure 3A-c,d). In contrast to sham-operated rats, remarkably increased immunoreactivity to Iba-1 was observed in both sides of L_4–6_ spinal cord in I/R group rats (Figure 3*B*, *P* < .05). In addition, the increased Iba-1 immunoreactivity prominent in the dorsal horns obviously inhibited in rats preconditioning with minocycline, TAK-242 or PDTC, however, increased in those receiving LPS (all *P* < .05). Figure 3*C* showed the quantitative measurement of the cells that were positive for Iba-1 staining recorded for each specimen in a blind fashion at 12 h and 36 h after I/R injury. The data showe that pretreatment with minocycline before ischemia significantly prevented the microglial activation and proliferation during a 36-h follow-up period after reperfusion (*P* < .01). Interestingly, neutralizing function against TLR4 and NF-κB by intrathecal infusion of TAK-242 and PDTC also significantly attenuated the microglial activation indicated by the decreased number of Iba-1 positive cells (*P* < .01). In contrast, many more Iba-1 positive cells were observed in spinal cord of rats pretreated with LPS at the same time point (*P* < .01). There were no significant differences between the groups of I/R + M, I/R + T and I/R + P at above time points (*P* > .05).

### Effects of I/R on expression and colocalization of TLR_4_ and microglial marker Iba-1 in the spinal cord

**Figure 4:**
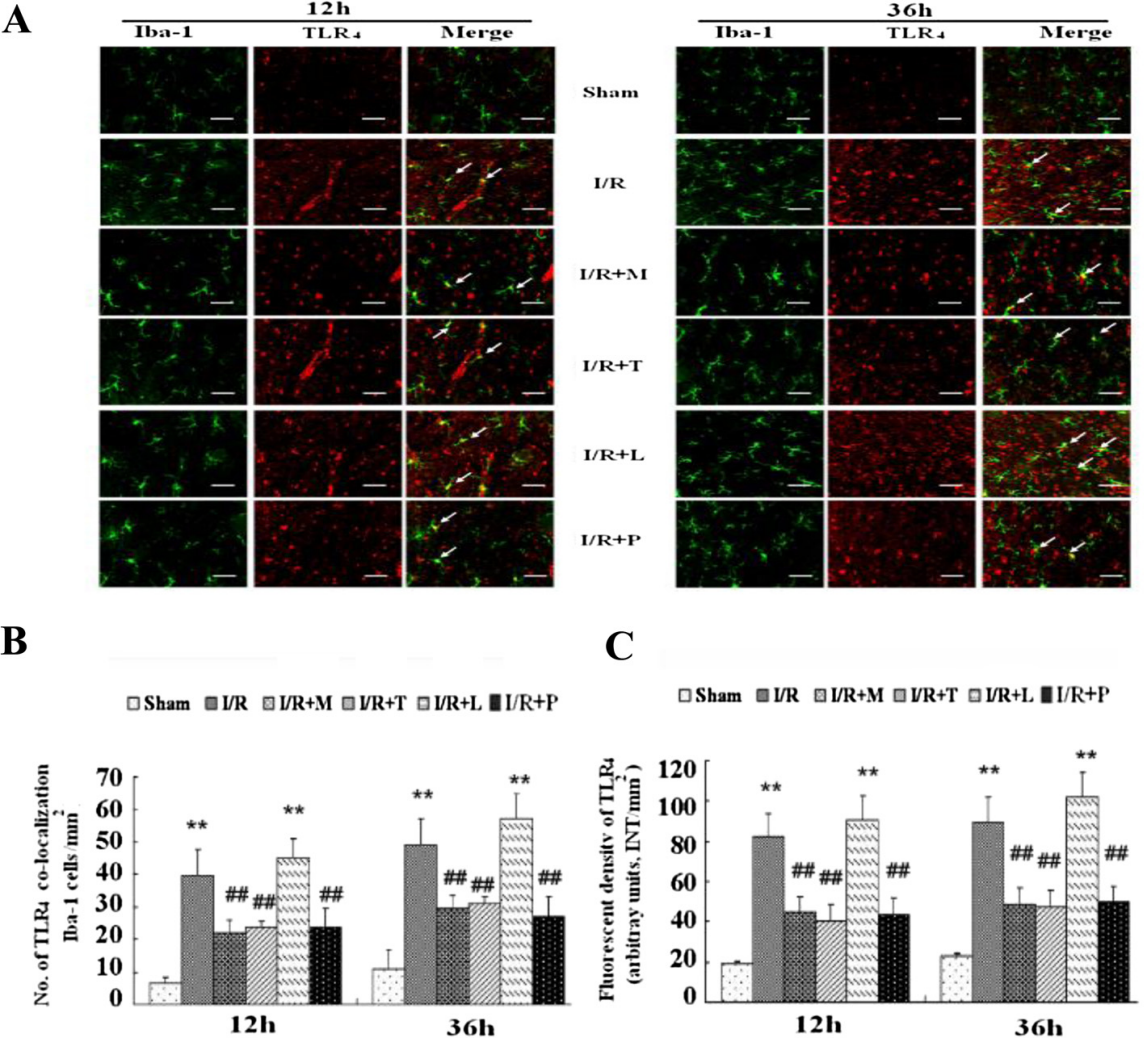
**Double immunostaining of microglial cells with its membrane-bound receptor TLR**_**4 **_**after spinal cord ischemia reperfusion (I/R) injury. (A)** Representative micrographs show the cellular location of Toll-like receptor (TLR_4_; red) with antibodies against microglial specific marker (Iba-1; green) at 12 h and 36 h after I/R injury. Arrows delineate co-localization. Scale bars are 100 μm. **(B)** Histogram for quantification of co-localized cells (cells with yellow signals). **(C)** Quantification of the TLR_4_ immunoreactivity is presented as average of three fluorescence intensity (FI) of three independent experiments. Double immunohistochemistry showed TLR_4_ was highly expressed on spinal microglial after I/R injury. The rats pretreated with minocycline, TAK-242, PDTC were suggested significantly decreased TLR_4_ immunoreactivity after I/R and the number of TLR_4_-Iba-1 positive microglia, whereas above effects synthetically increased in rats receiving LPS. All data are presented as mean ± SEM (n = 8 per group). **P < .01 compared to Sham group; ^##^*P* < .05 compared to I/R group.

To investigate whether TLR_4_ was involved in I/R-induced microglial activation and to determine its function, we performed double immunofluorescent staining at 12 and 36 h after reperfusion. As shown in Figure 4*A*, TLR_4_ with the identical fluorescence label were colocalized with the distribution of Iba-1–positive microglia in I/R group rats, but not in sham-operated ones. Thus, the data confirmed that TLR_4_ was involved in microglial activation after I/R injury (*P* < .05). Pretreated with TAK-242 to neutralize function against TLR_4_ markedly inhibited Iba-1 immunoreactivity, whereby up-regulation with TLR_4_ expression by LPS largely increased Iba-1 immunoreactivity (Figure 4*A*, *P* < .05). Similar quantification of TLR_4_ co-localization in Figure 4*B* and TLR_4_ immunoreactivity in Figure 4*C* confirmed that TLR_4_ was necessary for the I/R-induced up-regulation of Iba-1 expression in activated microglial.

Additionally, there were no significant differences in the amount of double-labeled cells, Iba-1 or TLR_4_ immunoreactivity betweens the groups of I/R + M, I/R + T and I/R + P at above time points (Figure 4*B* and *C, P* > .05)

### Effects of I/R on Neuronal reaction in the spinal cord

**Figure 5:**
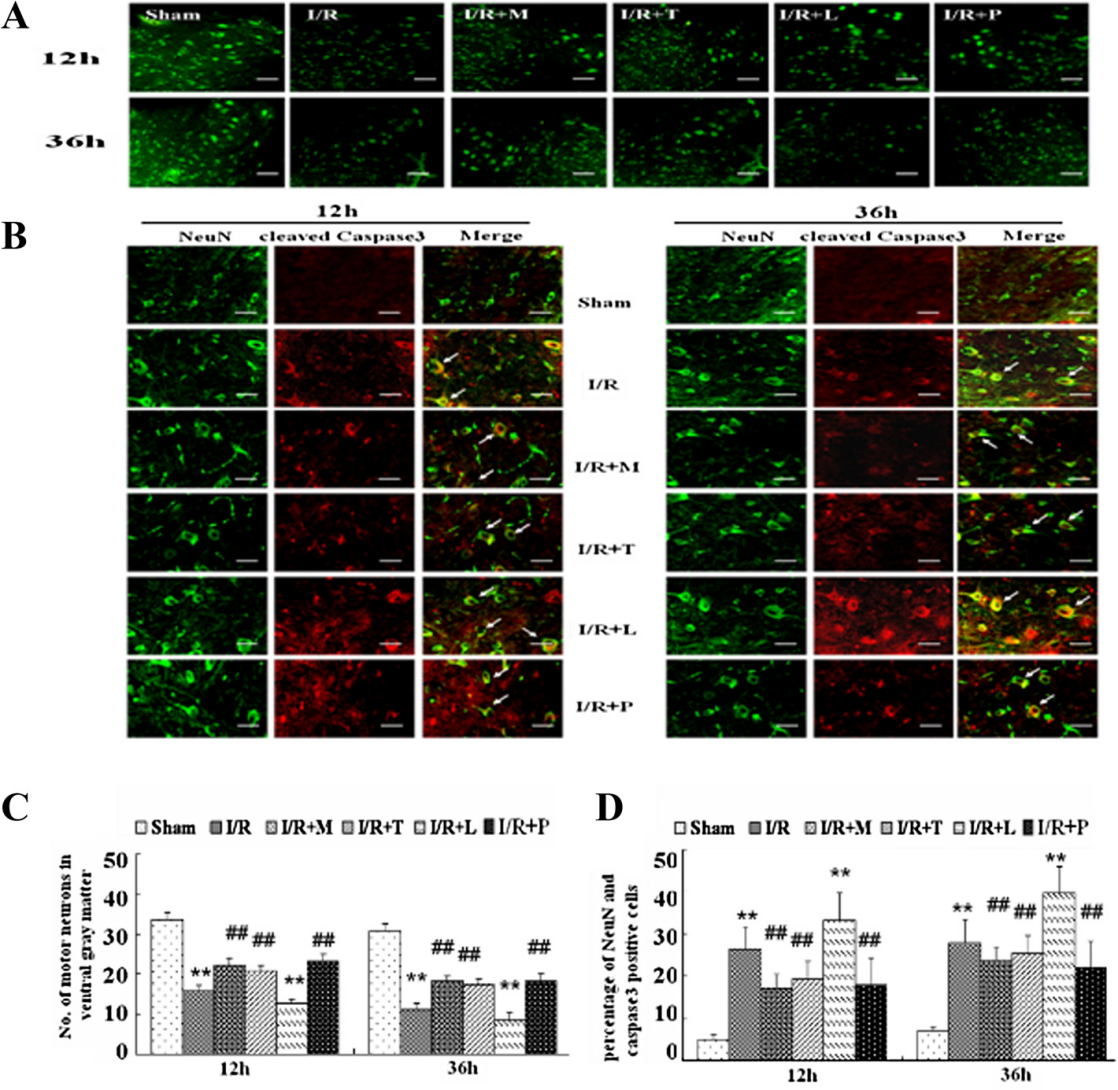
**Alterations in Neuronal reaction after spinal cord ischemia reperfusion (I/R) injury. (A)** Effects on spinal immunoreactivity to neuronal marker NeuN in laminae IX of ventral gray matter after I/R injury. Scale bars are 200 μm. **(B)** Representative immunohistochemical localization of neurons (NeuN; green) and cleaved caspase3 (red) in laminae IX of spinal cord at 12 h and 36 h after I/R injury. Arrows delineate co-localization. Scale bars are 100 μm. **(C)** Quantification of the NeuN-positive neurons in laminae IX of ventral gray matter is presented as average of three independent experiments. **(D)** Histogram of the values for co-localized cells (cells with yellow signals) as the percent of the total NeuN-positive cells in laminae IX of ventral horn. Immunohistochemistry showed I/R led to the decrease in neuronal number of both spinal ventral horns and increase percentage of NeuN-cleaved caspase3-positive cells, suggesting the loss of neurons partly as a result of apoptosis. Pretreated with minocycline, TAK-242, PDTC were showed had neuroprotective effects as the decreases in number of neuronal apoptosis, intrathecal injection with LPS abrogated above effects. All data are presented as mean ± SEM (n = 8 per group). **P < .01 compared to Sham group; ^##^*P* < .05 compared to I/R group.

To determine whether TLR_4_ was involved in I/R-induced inflammatory damage due to neurological deficit or neuronal apoptosis most likely, we performed double immunofluorescent staining of neuronal-specific marker neuronal nuclei (NeuN) and cleaved caspase3 at 12 and 36 h after reperfusion. As shown in Figure 5A, comparing with the sham group, there was remarkably decreased NeuN immunoreactivity observed in ventral gray matter of rats in I/R group (*P* < .05). In addition, the decreased NeuN immunoreactivity was greatly prevented in rats preconditioning with minocycline, TAK-242 or PDTC, nevertheless, synthesized in those receiving LPS (all *P* < .05). The quantifications of NeuN expressions were shown in Figure 5*C.*

Furthermore, double immunofluorescence in Figure 5*B* showed that abundant capase-3 positive neurons in spinal cord of rats in I/R group at both 12 h and 36 h, and the amount r of double-labeled neurons was significantly decreased in rats pretreated with minocycline, TAK-242 or PDTC (all P < .05 ). In contrast, the neurons had a higher density of co-staining with cleaved caspase3 in rats receiving LPS (P < .05). Quantification of the percentage of double-labels cells was shown in Figure 5D.

### Effects on TLR4 -mediated NF-κB/IL-1β signal pathway in spinal cord after I/R

**Figure 6:**
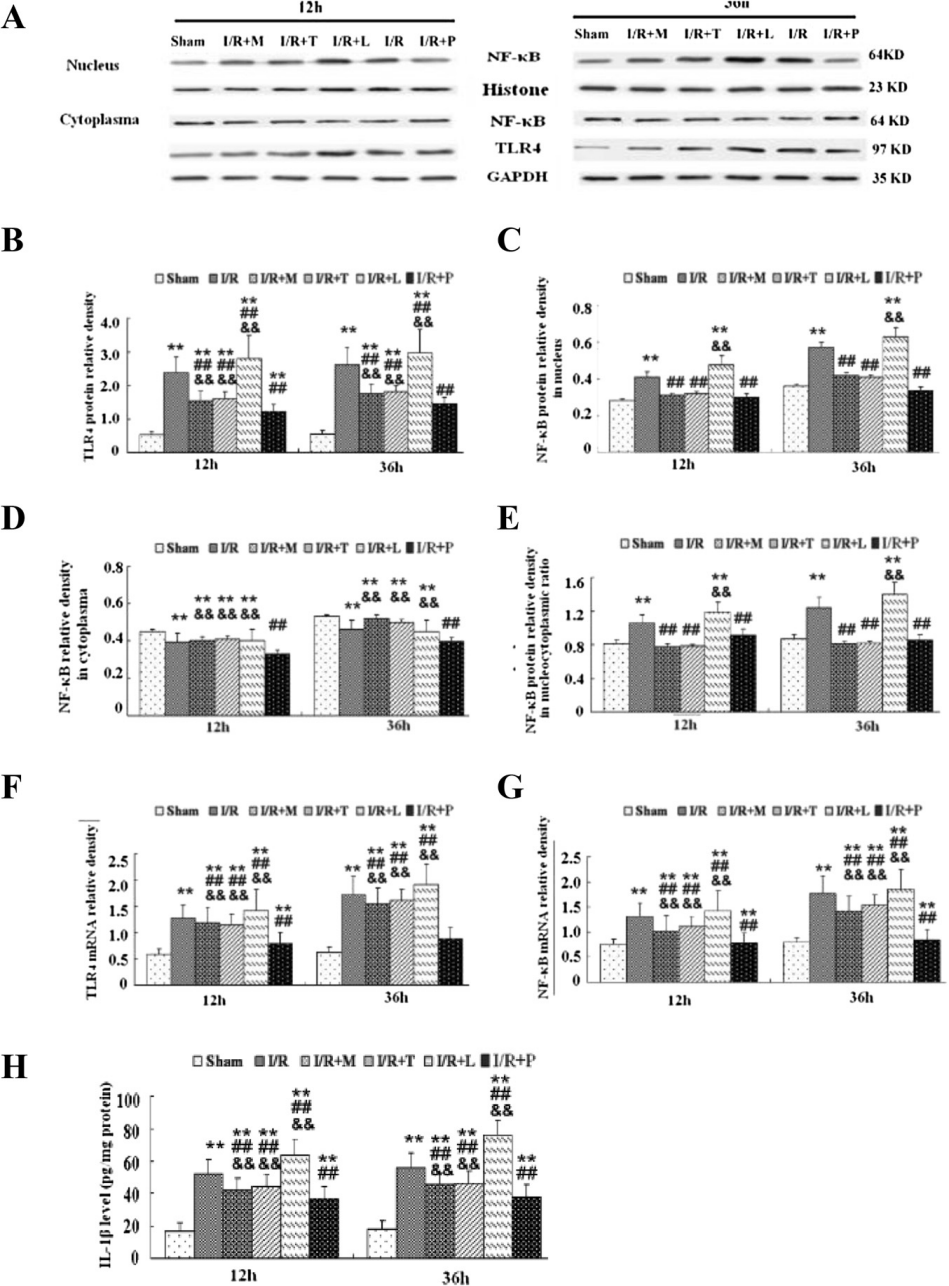
**Alterations in TLR**_**4 **_**and NF-**κ**B protein and mRNA expressions after spinal cord ischemia reperfusion (I/R) injury. (A)** Representative immunoblots were probed with antibody against TLR_4_ and NF-κB p65, while antibody against Histone and GAPDH served as loading control for the nuclear and cytoplasmic protein, respectively. (B-E) Quantification of the densities of TLR_4_**(B)**, NF-κB p65 bands in nuclear extracts **(C)**, cytoplasmic extracts **(D)** and calculated as nucleocytoplasmic ratio **(E)** in different protocol conditions. The protein expression is presented in relative units. **(F-G)** Real-time PCR analysis was performed in duplicate for TLR4 and NF-κB p65 under study and normalized to *GAPDH* mRNA. **(H)** Quantification data of IL-1β production in the spinal cord at 12 h and 36 h after I/R injury, as assessed by ELISA. Ordinate represents the mean integral density values (IDVs) ratios relative to the loading control. I/R caused significant increases in TLR_4_, nuclear and cytoplasmic NF-κB p65 expressions, as well as nucleocytoplasmic ratio at 12 h and 36 h after I/R after normalizing to Histone and GAPDH, consistent with results from real time-PCR. Intrathecal injection with minocycline, TAK-242 and PDTC prevented NF-κB p65 upregulation in nucleocytoplasmic ratio, as the upregulated expressions of NF-κB p65 in nucleus and comparable expressions in cytoplasma. Contrarily, intrathecal injection with LPS synergistically increased the activation. IL-1β content was changed accordingly with the mRNA and protein expressions of TLR_4_ and NF-κB by ELISA. All data are presented as mean ± SEM. **P < .01 compared to Sham group; ^##^*P* < .05 compared to I/R group; &&*P* < .05 compared to I/R + P group.

The expression of TLR_4_ was measured to further investigate its effect on microglial activation during I/R-induced inflammatory processes. As shown in Figure 6*A*, I/R injury induced markedly increases protein and gene expressions of TLR_4_ in comparison to sham group at 12 h and 36 h after injury (*P* < .05). Pretreated with minocycline, TAK-242 or PDTC prevented such increases, whereas stimulation of TLR_4_ using LPS up-regulated TLR_4_ expression synergistically (*P* < .05). The quantifications of TLR_4_ expressions were shown in Figure 6*B* and *F.*

**Figure 7:**
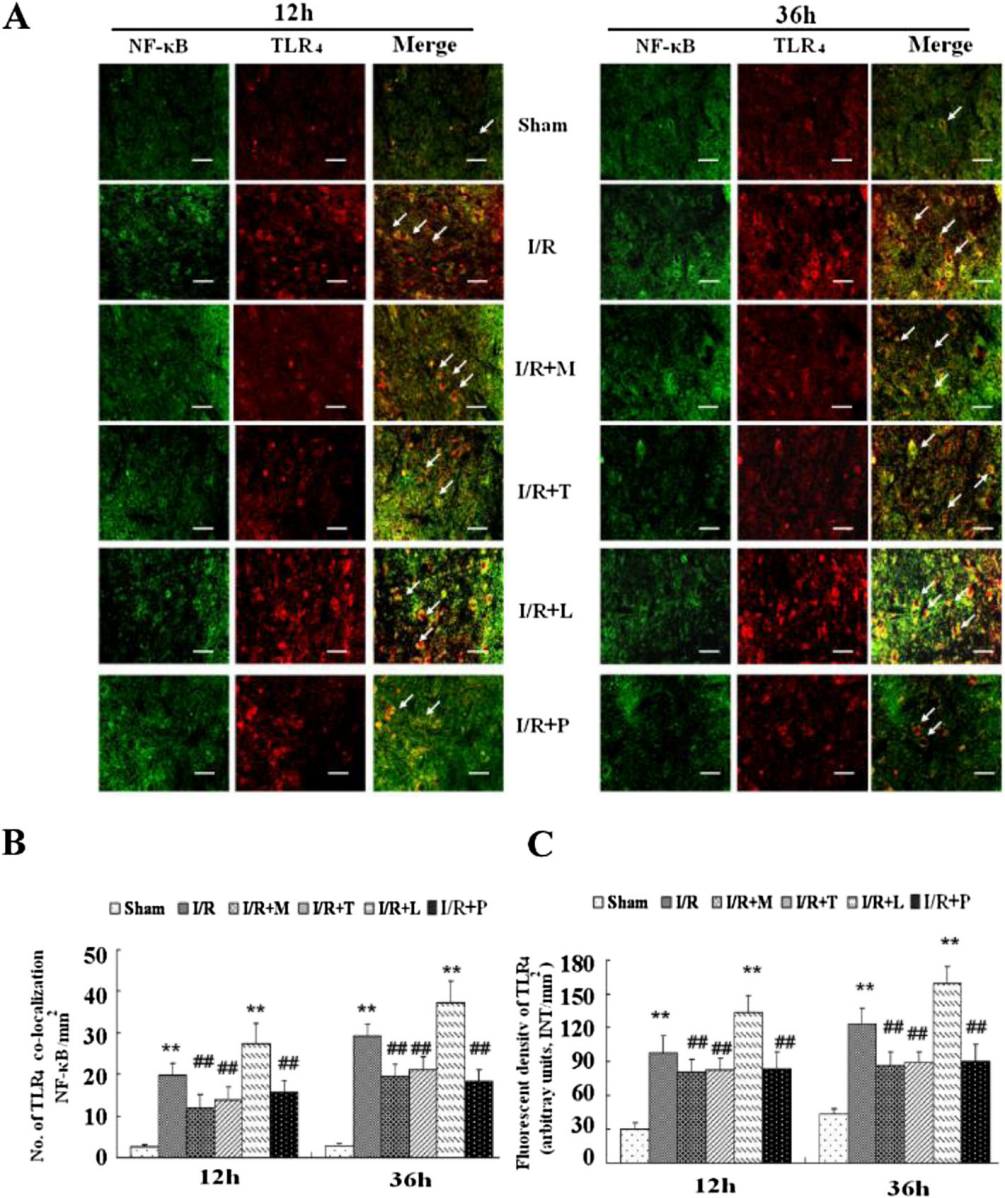
**Double immunostaining of TLR**_**4 **_**with transcription factor nuclear factor** κ**B (NF-**κ**B) after spinal cord ischemia reperfusion (I/R) injury. (A)** Representative micrographs show the colocation of TLR_4_ (red) and NF-κB (green) at 12 h and 36 h after I/R injury. Arrows delineate co-localization. Scale bars are 100 μm. **(B)** Histogram for quantification of co-localized cells (cells with yellow signals). **(C)** Quantification of the TLR_4_ immunoreactivity is presented as average of three fluorescence intensity (FI) of three independent experiments. Double immunohistochemistry showed NF-κB had similar profile as TLR_4_ immunoreactivity after I/R injury. The rats pretreated with minocycline, TAK-242, PDTC were suggested significantly decreased TLR_4_ immunoreactivity after I/R and the number of TLR_4_- NF-κB-positive cells, whereas above effects synthetically increased in rats receiving LPS. All data are presented as mean ± SEM (n = 8 per group). **P < .01 compared to Sham group; ^##^*P* < .05 compared to I/R group.

As shown in Figure 4*,* the data suggested that the number and dense of Iba-1-TLR4-postive punctuated dots in group pretreated with PDTC were comparably to those in groups receiving minocycline and TAK-242 at above time points (*P* > .05). Thus, we further examine whether NF-κB pathway was involved in the up-regulation of TLR_4_. The immunofluorescent stain and quantitative results in Figure 7*A* and *B* showed that I/R led to a significant increase in NF-κB expression. Presence of LPS during I/R synthetically up-regulated NF-κB, whereas this effect was abrogated with TAK-242 treatment which inhibits TLR_4_ function (*P* < .05). Furthermore, pretreated rats with minocycline, TAK-242 or PDTC decreased the number of double-labeled cells (Figure 7*B, P* > .05) and TLR_4_ immunoreactivity (Figure 7*C, P* > .05).

Next, we evaluated the nuclear and cytoplasmic NF-κB p65 expressions respectively to investigate the cellular mechanisms which might attenuate the development of I/R. Quantification of NF-κB p65 after normalized to Histone and GAPDH in each sample were shown in Figure 6 C and D. The data demonstrated that I/R significantly activated NF-κB p65 in nuclear fractionation. Specifically, NF-κB p65 in I/R group increased by 1.105-fold and 1.234-fold relative to *Histone* at 12 and 36 h after injury respectively (Figure 6*C, P* < .05), whereas the expression in cytoplasmic fractionation was decreased by 0.866-fold and 0.867-fold relative to *GAPDH* (Figure 6*D, P* < .05). Moreover, the results were further supported by quantification of NF-κB p65 in nucleocytoplasmic ratio shown in Figure 6*E.* The results showed that nucleocytoplasmic ratio in I/R group at 12 h and 36 h, respectively increased by 1.297-fold and 1.428-fold relative to sham group (*P* < .05), consistent with real time-PCR results (Figure 6*G*). Significantly, pretreated with minocycline and TAK-242 only prevented these increase in nucleus (Figure 6*C*, *P* < .05), whereas PDTC treatment prevented these increase in both nucleus and cytoplasma (Figure 6*C* and *D, P* < .05).

Furthermore, to verify the inflammatory signal downstream activation, we detected the inflammatory cytokine IL-1β activation by ELISA. The data presented that IL-1β was activated along with the activation of TLR_4_ and NF-κB (Figure 6H, *P* < .05). Pretreated with minocycline, TAK-242 or PDTC inhibited such activation, whereas stimulation of TLR_4_ using LPS enhanced IL-1β content synergistically (*P* < .05).

## Discussion

Ischemia/reperfusion (I/R) injury of the spinal cord after an operation on the thoracic aorta is an unpredictable, however, disastrous complication. To the best of our knowledge, the current study is the first to demonstrate activation of TLR_4_ in microglia and we show TLR_4_ is participates in inflammatory reactions in the blood–spinal cord barrier (BSCB) after I/R injury. Early and sustained microglial activation can be determined by the mRNA and protein expression of the microglial surface markers, Iba-1. The membrane-bound receptor TLR_4_ is also overexpressed in the spinal cord but not in sham-operated rats from 12 to 36 h after I/R injury. In addition, intrathecal administration of minocycline for 3 days before ischemia showed obvious protective effects in the form of a decrease in I/R-induced microglial activation, in BSCB disruptions, and in the production of proinflammatory cytokines. Results from this study also demonstrated that when administered in this preemptive manner, intrathecal infusion of a TLR_4_ receptor inhibitor, TAK-242, exhibits protective effects similar to minocycline pretreatment results, whereas intrathecal injection of LPS, a TLR_4_ agonist, exacerbates deleterious effects. Given that NF-κB is known to be activated in the presence of TLR_4_, it is expected that the NF-κB inhibitor,PDTC inhibit I/R-induced microglial activation via upregulating TLR_4_.

The I/R model used and the period observed were referred to previous studies, in which the evolution of inflammatory cytokine activation in the spinal cord peaks between 24 and 48 h after reperfusion [5,6,16]. This model is a reliable and stable animal model for studying neuroprotective manipulations and molecular mechanisms in the spinal cord. BSCB consists of a continuous capillary endothelium with tight junctions between its cells, surrounded by astrocytes and perivascular microglia. The intact barrier can prevent vasogenic edema and pathological effects on CNS by restricting access of molecules and cells to the spinal cord under conditions of stroke and I/R [17,18]. It is generally believed that inflammatory factors play a critical role in leakage of BSCB as a result of aberrant vascular permeability due to dissociation of zonula occludens-1 (ZO-1) from the cytoskeletal complex and due to an increased level of matrix metalloproteinases (MMPs) and tumor necrosis factor α (TNF-α) [5,6,17-19]. As demonstrated in our previous study, BSCB permeability is altered in the course of spinal cord I/R injury, and these changes could be measured using extravasation of EB dye [5,6]. This damage may caused by inflammatory processes and it may further exacerbate inflammation. Recent evidence support the notion that microglia, components of the immune system are activated and play an important role in the initiation phase of I/R-induced neurodegenerative and inflammatory processes. When triggered by peripheral inflammation or nerve injury, spinal microglia can be rapidly activated and can respond to the neurotransmitters released by central terminals of primary sensory neurons, such as glutamate, substance P, and adenosine triphosphate. After that, the release from activated glial cells of a series of growth factors, chemokines, regulatory cytokines as well as free radicals and other toxic mediators such as IL-1β, TNF-α, prostaglandin E_2,_ and reactive oxygen species (ROS), results in activation of rapid signal transduction cascades leading to either survival or death of neurons [9,17]. Representative micrographs of ionized calcium–binding adaptor molecule 1 (Iba-1) are commonly used to quantify activated microglia. Upon activation, microglial cells transform from the ramified shape to rounded (amoeboid) macrophage-like morphology [20]. There are significantly greater numbers of Iba-1–positive cells in the I/R group both at 12 and 36 h in comparison to the lower numbers in the sham group. Furthermore, the colocalization with TLR_4_ according to double-immunofluorescence analysis confirmed that TLR_4_ is indeed upregulated in activated microglial cells in injured regions of the spinal cord.

There are substantial protective effects of minocycline (a member of the tetracycline antibiotic family), which prevent microglial activation and generation of glutamate, IL-1β, and nitric oxide (NO) [21]. To explore the role of microglia in BSCB disruption after I/R injury, minocycline was infused intrathecally during 3 days before the surgical procedure in the present study. We also found that inhibition of microglial activation by minocycline is accompanied by a decreased number of Iba-1–positive cells.

Blamire et al. [22] and van Vliet et al. [23] examined the role of proinflammatory cytokines in BSCB leakage. These investigators reported that increased plasma levels of inflammatory cytokines (such as IL-1β and IL-6) in residual microglia (where disruption of the blood–brain barrier occurred) might also be responsible for BSCB leakage. At the same time, we observed another protective role of minocycline: it seems to attenuate (1} the BSCB disruption as measured by EB extravasation and (2} an increase in spinal water content. Our data show that minocycline decreases the number of Iba-1–positive cells at 12 and 36 h after reperfusion and attenuates upregulation of the proinflammatory molecules IL-1β and NF-κB in the spinal cord. These findings were corroborated by previous studies not only in experiments with ischemia [3,4] but also in studies of systemic proinflammatory states [9,23,24]. These observations suggest that activated microglia may potentiate damage to BSCB components, which is caused at least in part by proinflammatory cytokines.

Previously, we elucidated the molecular mechanisms underlying inflammatory and immunological processes of microglial activation after I/R. Upon activation, microglial cells start to express TLR_2–4_ on their surface [11,14]. Among diverse TLRs, it has been reported that TLR_4_ enables microglia to induce immune and inflammatory responses and to release massive amounts of proinflammatory cytokines via activation of NF-κB, once TLR_4_ binds to its endogenous or exogenous ligands. Studies show that TLR_4_-deficient mice display significantly attenuated behavioral hypersensitivity and are characterized by weaker spinal glial activation and lowered release of proinflammatory cytokines [8,12,14]. There is usually no viral or bacterial infection in I/R models; recent research supports the notion that upregulated expression of TLR_4_ is implicated in activation of microglia in cerebral ischemia models [8,16]. Nonetheless, there is still debate and controversy regarding the role of microglial TLR_4_ in the spinal cord during the earliest stage (<3 days) after I/R, where TLR_4_ must be involved in various signal transduction pathways. Our results show that early, robust, and sustained microglial activation after I/R injury is characterized by a marked long-term induction of the TLR_4_ expression at protein and mRNA levels, matching the pattern of immunostaining with Iba-1. In parallel, cytokines IL-1β are released in the spinal cord between 12 and 36 h after reperfusion, the finding that is consistent with the above reports. We also confirmed the specificity and the role of TLR_4_ in the microglial activation and in inflammatory reactions induced by intrathecal infusion of TLR_4_ receptor antagonists or agonists. We found that downregulation of TLR_4_ receptor by TAK-242 lowers the amount of resident microglial activated cells, decreases levels of NF-κB translocation, and consequently downregulates the cytokine IL-1β in the spinal cord between 12 and 36 h after reperfusion. Because TLR_4_ is a known specific receptor for Gram-negative bacterial components (LPS), the rats treated with LPS showed aggravated inflammatory processes, and BSCB disruption occurred in the I/R region, in keeping with the pattern of upregulated TLR_4_.

In view of the pivotal role of TLR_4_ in microglial activation in the initial phase of I/R injury, the question arises how TLR_4_ mediates microglial activation in spinal cord I/R injury. After activation of the TLR_4_ signaling pathway, NF-κB relocates to the nucleus and regulates expression of target inflammatory genes via modulation of both MyD_88_ or MyD_88_ adaptor–like adaptor protein and TIR domain–containing adaptor [12,16,25]. With double immunofluorescence in present study, we provided evidence that NF-κB signaling pathway activated by TLR_4_ receptor played important roles in regulating immune and inflammatory responses after I/R. We detected greatly upregulation of NF-κB and IL-1β (at the mRNA and protein level) in spinal cords limited to the ischemic region between 12 and 36 h after I/R injury and was accompanied with a selective increase in TLR_4_ expression. Pyrrolidine dithiocarbamate (PDTC) is a low-molecular-weight thiol compound, was initially regarded as a potent inhibitor of NF-κB by inhibiting factor I-κB phosphorylating, thus preventing the dissociation of the NF-κB -IκB complex and interfering with the generation of proinflammatory cytokines [26-28]. It has been reported that intrathecal PDTC can delay and reverse mechanical allodynia in several neuropathic pain conditions [27]. Similarly, in present study, intrathecal infusion of PDTC was observed to have suppressive effects on mechanical allodynia, BSCB dysfunction and microglial activation as well as up-regulated TLR_4_ mRNA and protein expressions in spinal cord, suggesting that the activity of NF-κB pathway could regulate spinal cord I/R injury via regulating TLR_4_.

Neurons play very important roles in the nervous system, involved in the processes of memory, sense and behavior. A result of I/R-induced neurological deficit was partly contributed to apoptosis of neurons in spinal cord as reported in our previous studies [2,5,6]. Our data of NeuN immunoreactivity sufficiently evidenced I/R lead to a decrease neuron number in ventral gray matter and increase percentage of double-labeled cells with cleaved capase3, which referred as a mark of apoptosis. Meanwhile, microglia may release various factors to support and guide the transfer of neurons, participate in neurons repair and regeneration [29,30]. IL-1β is one of the final inflammatory molecules produced in TLR_4_ signaling pathway, which elicits a cascade of activation of cytokines and multiple biological effects [22]. In a variety of inflammatory conditions, reasonable upregulation of IL-1β has been shown to limit extreme inflammatory responses, with a possibility of rapidly activated phagocytosis of dead or dying cells to prevent a release of a cascade of proinflammatory cytokines and to resolve inflammation. Nevertheless, excess IL-1β can significantly worsen inflammation and tissue injury [9,13,22]. Thus, these signals should be regulated very tightly to balance proinflammatory and anti-inflammatory pathways. Brikos and colleagues found that the cytoplasmic portion of TLRs, called the Toll/IL-1 receptor (TIR) domain is highly similar to that of the IL-1 receptor family [1]. Our study confirmed that IL-1β expression and loss and apoptosis of neurons triggered by TLR4/ NF-κB signal in spinal cord were strongly increased by intrathecal infusion of LPS; in contrast, the expression levels were much lower in rats treated with minocycline, TAK-242 or PDTC, indicating that there might be a positive feedback circuit. In other words, the expression of inflammatory cytokines is regulated by TLR_4_ in microglia, and in turn, inflammatory cytokines can cause microglial activation by amplifying and maintaining inflammatory response via the TLR_4_ pathway. This notion also offers a probable reason for the difficulty in developing an effective therapy for spinal cord injury–related complications. Interestingly, the similar findings have been reported by Bell at al in a mice model of aortic cross-clamping, recently [31]. That study showed that inhibition of TLR_4_-mediated microglial activation may be a major mechanism of neuroprotection associated with the anti-inflammatory effects. Regarding the controversial effects of microglial activation via its plenty of membrane-bound receptors for spinal cord recovery under different stimuli, further studies would be required to clarify the complicated role of microglial TLR_4_ signaling in models of I/R injury. As potential therapeutic modalities, minocycline, TAK-242 and PDTC would need further research, including safety testing; for example, their effects on learning and memory are unknown.

Based on our results, we conclude that inhibition of microglial activation and proliferation via the TLR_4_–microglia–NF-κB/IL-1β pathway result in protective effects in some neurological deficits, hence, fortifying BSCB integrity, and reducing spinal cord swelling.

## Conclusion

Taken together, our data provide evidence to support the involvement of TLR_4_ signaling in modulation of microglia activation in spinal cord I/R injury. We also suggest that TLR_4_–microglia– NF-κB/IL-1β pathway as a positive feedback loop in the spinal dorsal horn after I/R injury; this pathway is capable of exacerbating inflammatory reactions and BSCB dysfunction. It could offer new therapeutic targets for managing severe and persistent complications of spinal cord injury caused by I/R.

## Materials and methods

### Experimental animals

All experimental procedures were approved by the Ethics Committee of China Medical University and were in compliance with the *Guide for the Care and Use of Laboratory Animals* (U.S. National Institutes of Health publication No. 85–23, National Academy Press, Washington DC, revised 1996). Male Sprague–Dawley rats, weighting 200–250 g were used in this study. All rats were maintained under standard condition throughout the experimental period. Either motor or sensory dysfunction was observed in rats that intrathecally received minocycline, lipopolysaccharide (LPS), TLR4 inhibitor (TAK-242), pyrrolidine dithiocarbamate (PDTC) or saline before the induction of ischemia.

### Experimental I/R spinal cord injury

The spinal cord I/R model was induced by occlusion of the aortic arch for 14 minutes, as previously reported [16].All rats were anaesthetized with intraperitoneal injection of 4% sodium pentobarbital at an initial dose of 50 mg/kg. Lung ventilation was achieved using a mouse ventilator (Hollinston, MA) with endotracheal intubation. Body temperature was continuously monitored with a rectal probe and was maintained at 37.5 ± 0.5°C with the aid of a heated operating table. A catheter was inserted into the left carotid artery and into the tail artery to measure proximal and distal blood pressure (Spacelabs Medical Inc., Redmond, WA, USA). Under direct visualization, the aortic arch was cross-clamped between the left common carotid artery and the left subclavian artery. A catheter was inserted into the left carotid artery and into the tail artery to measure proximal and distal blood pressure. Ischemia was confirmed as a 90% decrease in flow measured at the tail artery by a laser Doppler blood flow monitor (Moor Instruments, Axminster, Devon, UK) for 14 min, after which, the clamp was removed and 36 h of reperfusion took place. Sham operation rats underwent the same procedure, but no occlusion of the aortic arch was performed.

### The experimental protocol

One hundred twenty rats were randomly assigned to 1 of 6 groups by means of a random number table: the I/R group (n = 24), I/R + minocycline (I/R + M) group (n = 24), I/R + lipopolysaccharide (I/R + L) group (n = 24), I/R + TLR4 inhibitor (TAK-242, I/R + T) group (n = 24), I/R + pyrrolidine dithiocarbamate (PDTC, I/R + P) group (n = 24) or the sham group (n = 24). Spinal cord I/R injury was induced by occlusion of the aortic arch for 14 min, whereas the aorta was exposed, but without occlusion in the sham group. In all groups, we performed intrathecal infusion of 10 μL normal saline, 10 nmol/μL minocycline (10 μL, Nichiiko, Toyama, Japan), 1 nmol/μL LPS (10 μL, Sigma; E. coli 011:B4), 10 nmol/μL TAK-242 (10 μL, EMD, Millipore; CAS 243984-11-4), 100 pmol/μL PDTC (10 μL, Sigma-Aldrich, Mo;71935) or 10 μL of saline respectively, continuously for 3 days before the surgical operation. The rats were euthanized 12 and 36 h after the surgical procedure. At each time point, animals were anesthetized with an overdose of pentobarbital and the L_4–6_ segments of spinal cords were rapidly collected for analysis because of their vulnerability to ischemic injury.

### Behavioral analysis

To quantify mechanical allodynia, the withdrawal threshold of a hind-limb paw was assessed using von Frey filaments (Stoelting Co., Wood Dale, IL, USA) and the Dixon up–down method as described by Chaplan and coworkers [32]. All behavioral tests were performed before the surgical procedure (baseline) and at 12-h intervals during a 36-h observation period by an observer who was blinded to the experimental procedures.

### Measurement of spinal cord edema

Water content of the spinal cord was measured by means of the wet–dry method as quantitative measurement of edema, as reported previously [5,6]. The percent water content was calculated using the following formula: %H_2_O = (wet weight − dry weight) × 100/wet weight.

### Measurement of Evans blue extravasation

After survival of rats for 12 and 36 h, Evans blue (EB) content and EB fluorescence were used for quantitative and qualitative analysis of BSCB disruption after spinal cord I/R injury, as described previously [5,6]. Briefly, EB at 30 g/L (45 mg/kg; Sigma) was slowly intravenously injected into the tail vein 60 min before euthanasia. After being adequately perfused with saline under deep anesthesia, the L_4–6_ segment was removed and soaked in methanamide for 24 h (60°C) and then centrifuged. EB content was measured as absorbance of the supernatant at 632 nm on a microplate reader (BioTek, Winooski, VT) and calculated as the amount of EB per wet tissue weight (μg/g). For measurement of the fluorescence, the tissue was fixed in 4% paraformaldehyde, sectioned (10 μm), and kept frozen and sealed in a light-tight container. EB staining was visualized using a BX-60 (Olympus, Melville, NY) fluorescence microscope (green filter). Percentage of recognized area (fluorescence intensity above the threshold) referred to the whole image area was performed using Image J software (NIH Image, Bethesda, MD).

### Iba-1 immunoreactivity

Microglia were stained using an antibody against the microglial marker, ionized calcium–binding adaptor molecule 1 (Iba-1) as described previously [17,32]. Briefly, the sections were firstly blocked with 10% bovine serum albumin for 1 h at room temperature. After that, the sections were incubated with a primary rabbit anti–Iba-1 antibody (1:800: Wako, 019–19741) at 4°C overnight. After incubation with an Alexa 488–conjugated donkey anti–rabbit IgG antibody (1:500; Molecular Probes, Rockford, USA) for 1 h, the stained sections were examined under a microscope (Carl Zeiss Axio Observer Z1, Jena, Germany) determined the number of immunoreactive cells in the medial superficial dorsal horn (laminae I–III). Nonspecific staining was determined by omitting the primary antibody. The data were calculated as average numbers of positive cells per area of a spinal section ± standard error of the mean (SEM).

### Double immunofluorescence

Double immunofluorescence analysis was carried out to confirm the expression of TLR4 in microglia and explored the relationship with NF-κB signal pathway and neuroapoptosis after I/R [5,32]. Briefly, spinal cord was fixed and sectioned into 10-μm slices with a Leica CM3050 S cryostat. The sections were blocked with 10% bovine serum albumin (BSA) for 1 h at room temperature and incubated overnight at 4°C with the primary antibodies: mouse anti-TLR4 (1:100, Abcam), mouse anti-cleaved caspase3 (1:400, Cell signal technology), rabbit anti-Iba-1 antibody (1:800, Wako), rabbit anti-NF-κB p65(1:500, Abcam), rabbit anti-NeuN (1:800, Abcam). After incubation with Alexa 594-conjugated donkey anti-mouse IgG (1:500, Molecular Probes) and Alexa 488-conjugated donkey anti-rabbit IgG (1:500, Molecular Probes) for 2 h at room temperature. Each of the steps above was followed by four rinses 5–10 min each in PBS containing 10% BSA and 0.25% Triton X-100. Images were captured using a Leica TCS SP2 (Leica Microsystems, Buffalo Grove, IL, USA) laser scanning microscope and photographed by the attached digital camera to determine the number of immunoreactive cells. Nonspecific staining was determined by omitting the primary antibody. The data were expressed as numbers of positive cells/area/spinal section ± standard error mean (SEM).

### Measurement of IL-1β using ELISA

The spinal cord was collected and homogenized, followed by centrifugation. The IL-1β content was determined using an ELISA kit (R&D Systems, Minneapolis, MN, US). According to the manufacturer’s instructions, absorbance (A) was quantified at λ = 450 nm. The IL-1β content of each sample was calculated based on the standard curve, and IL-1β concentration was expressed in pg/mg protein.

### Western blots

The protein expression of TLR4 and NF-κB p65 in spinal cord tissue was determined using Western blotting analysis. The rats’ spinal cords were homogenized and nuclear and cytoplasmic extracts was purified from each specimen by using Nucleoprotein and cytoplasmic protein extraction kit according to the manufacturer’s instructions (KGP-150; KangChen, Shanghai, China). The antibodies used in this experiment were mouse monoclonal anti-TLR4 (Abcam), rabbit polyclonal anti–NF-κB p65 (phospho S536, Abcam), mouse monoclonal anti–Histone (Abcam) and anti-mouse GAPDH (dilution 1:10,000, Abcam) overnight on a shaker at 4°C.After three washes with TBS-0.1% Tween, the membranes were incubated with horseradish peroxidase-conjugated secondary antibodies(Bioss, Beijing, China) for 1 h. Semiquantitation of scanned images was performed using Quantity One software (Bio-Rad Laboratories, Milan, Italy).

### Real-time PCR

Quantitative real-time PCR was performed as described previously [20,21]. Total RNA was extracted from L_4–6_ spinal cord tissue using the TRIzol reagent (Invitrogen–Life Technologies), following the manufacturer’s instructions. PCR was performed as described previously using a SYBR Green SuperMix-UDG and was conducted on a Prism 7000 detection system (Applied Biosystems, Foster City, CA). The following primers were used; TLR_4_ (NM_0191178, 127 bp): forward 5′-GGATGATGCCTCTCTTGCAT-3′, reverse 5′-TGATCCATGCATTGGTAGGTAA-3′; NF-κB (HL26267H): forward 5′-CTTCTCGGAGTCCCTCACTG-3′, reverse 5′-CCAATAGCAGCTGGAAAAGC-3′; and *GAPDH* (HNM_023964H, 238 bp): forward 5′-AGAAGGCTGGGGCTCATTTG-3′, reverse 5′-AGGGGCCATCCACAGTCTTC-3′. Amplification was performed using the following cycling conditions: 50°C for 2 min (uracil-DNA glycosylase incubation), 95°C for 10 min, followed by 40 cycles of denaturing at 95°C for 15 seconds and annealing at 60°C for 30 seconds. All reactions were performed in triplicate. Gene expression was calculated relative to the endogenous control samples (*GAPGH*) to obtain a relative quantity (RQ) value (2-ΔΔCt, where CT is the threshold cycle).

### Statistical analysis

All data were collected by investigators blinded to surgery status of the rats. The data were calculated as mean ± SEM and analyzed using the SPSS software (version 17.0, SPSS Inc., Chicago, IL, USA). The statistical data were processed with one-way analysis of variance (ANOVA) followed by Newman–Keuls *post hoc* analysis. Differences with a *P* value of <0.05 were considered statistically significant (Additional file 1: Figure S1).

## Abbreviations

I/R: Ischemia-reperfusion; BSCB: Blood-spinal cord barrier; TLR4: Toll-like receptor 4; MyD88: Myeloid differentiation factor _88_; TRIF: TIR domain-containing adaptor-inducing IFN-β; LPS: Lipopolysaccharide; Iba-1: Ionized calcium–binding adaptor molecule 1; NF-κB: Nuclear factor kappa-B; IL: Interleukin; EB: Evan’s Blue; ROS: Reactive oxygen species; NO: Nitric oxide; MMPs: Matrix metalloproteinases; Iba-1: Ionized calcium–binding adaptor molecule 1; NeuN: Neuronal nuclei.

## Competing interests

The authors declare that they have no competing interests.

## Authors’ contributions

X-QL and BF participated in the animals’ care and made all the animal models. X-QL, JW and W-FT participated in tissue preparation, and sectioning and performed most immunohistochemistry; X-QL, BF and W-FT performed western blotting assay and statistical analysis; HM involved in the guide of model design and study design; JW gave important directions to data analysis and manuscript writing. All authors read and approved the final manuscript.

## Supplementary Material

Additional file 1: Figure S1 Western blotting analysis of transcription factor NF-κB proteins in each group of spinal cords after I/R injury. (A) Representative immunoblots were probed with antibody against NF-κB p65, while antibody against Histone and GAPDH served as loading control for the nuclear and cytoplasmic fraction, respectively. (B-D) Quantification of the densities of NF-κB p65 bands in nuclear extracts (B), cytoplasmic extracts (C) and calculated as nucleocytoplasmic ratio (D) in different protocol conditions at 12h and 36h after I/R injury. The protein expression is presented in relative units. (E-F) Quantification of the densities of p-NF-κB p65 and p-I-κB in total protein, while anti-NF-κB p65 and anti-I-κB served as the corresponding loading controls at 12 and 36 h after injury. Ordinate represents the mean integral density values (IDVs) ratios relative to the loading control. The data are presented as mean ± SEM. **P < .01 compared to Sham group; ^##^P < .05 compared to I/R group;&& compared to I/R+P group. I/R caused significant increases in nuclear and cytoplasmic NF-κB p65 expressions, as well as nucleocytoplasmic ratio at 12h and 36 h after I/R after normalizing to Histone and GAPDH, respectively. The results were consistent with the changes of phosphorylation of NF-κB and I-κB in total protein of spinal cord’s homogenates. Intrathecal injection with minocycline, TAK-242 and PDTC attenuated I/R-induced NF-κB p65 activation manifested as the decreased nuclear NF-κB p65, phosphorylation of NF-κB p65 and I-κB in total protein as well as NF-κB p65 in nucleocytoplasmic ratio at both timepoints, whereas injection with LPS synergistically increased the activation. Click here for file
