# Supplementary figures and images for: Intrathecal antagonism of microglial TLR4 reduces inflammatory damage to blood–spinal cord barrier following ischemia/reperfusion injury in rats
Source: Mol Brain. 2014 Apr 21;7:28. doi: 10.1186/1756-6606-7-28 (PMC4022154; doi:10.1186/1756-6606-7-28)

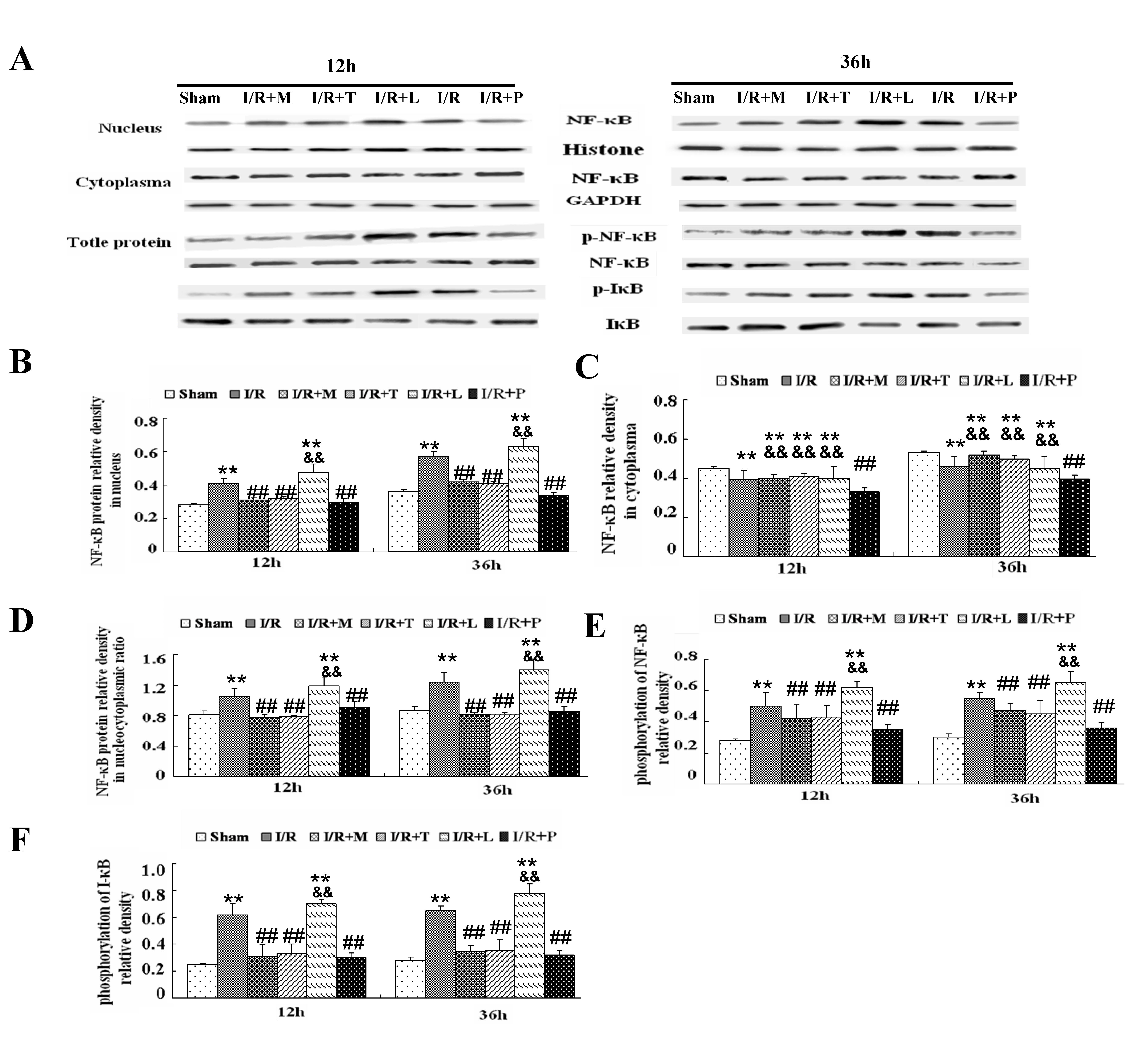

Supplement: Additional file 1: Figure S1 — Western blotting analysis of transcription factor NF-κB proteins in each group of spinal cords after I/R injury. (A) Representative immunoblots were probed with antibody against NF-κB p65, while antibody against Histone and GAPDH served as loading control for the nuclear and cytoplasmic fraction, respectively. (B-D) Quantification of the densities of NF-κB p65 bands in nuclear extracts (B), cytoplasmic extracts (C) and calculated as nucleocytoplasmic ratio (D) in different protocol conditions at 12h and 36h after I/R injury. The protein expression is presented in relative units. (E-F) Quantification of the densities of p-NF-κB p65 and p-I-κB in total protein, while anti-NF-κB p65 and anti-I-κB served as the corresponding loading controls at 12 and 36 h after injury. Ordinate represents the mean integral density values (IDVs) ratios relative to the loading control. The data are presented as mean ± SEM. **P < .01 compared to Sham group; ##P < .05 compared to I/R group;&& compared to I/R+P group. I/R caused significant increases in nuclear and cytoplasmic NF-κB p65 expressions, as well as nucleocytoplasmic ratio at 12h and 36 h after I/R after normalizing to Histone and GAPDH, respectively. The results were consistent with the changes of phosphorylation of NF-κB and I-κB in total protein of spinal cord’s homogenates. Intrathecal injection with minocycline, TAK-242 and PDTC attenuated I/R-induced NF-κB p65 activation manifested as the decreased nuclear NF-κB p65, phosphorylation of NF-κB p65 and I-κB in total protein as well as NF-κB p65 in nucleocytoplasmic ratio at both timepoints, whereas injection with LPS synergistically increased the activation. [file 1756-6606-7-28-S1.tiff]
